# Supplementary material for: Salinity tolerance, hyposaline stress recovery, and survival of the nemertean worm, Carcinonemertes carcinophila (Nemertea) in relation to its host, the Atlantic blue crab, Callinectes sapidus
Source: PLoS One. 2025 Jul 9;20(7):e0326493. doi: 10.1371/journal.pone.0326493 (PMC12240321; doi:10.1371/journal.pone.0326493)
Supplement: Attachment — Submitted filename: Response to Reviewers.docx [file pone.0326493.s002.docx]

August 29, 2025

Guillaume Marchessaux, Ph.D.
Academic Editor
PLOS ONE

Dear Dr. Marchessaux:

We have accepted nearly all of the reviewer’s comments and provide our response below. We provide a rebuttal to a few specific comments, but we are in agreement for the most part. The reviewer’s comments are organized by order of appearance in the original text, and our responses are in blue text. We have also addressed the specific journal requirements with our responses in blue text as indicated.

Sincerely,

Jeff Shields, Alexandria Pomroy, and Alexandra Schneider

Reviewer:

This study investigates the impact of salinity on nemertean parasites within blue crabs, a species of significant ecological and economic value. Key strengths include its focus on host-parasite interactions and how environmental factors like salinity influence them, particularly in euryhaline organisms. The research also contributes to understanding parasite tolerance to hyposaline stress, which is important for predicting their distribution. Given blue crabs' commercial importance, the study's findings have applied relevance for fisheries management. The controlled microcosm experiments and the inclusion of behavioral observations, such as phototaxis and probe response, enhance the study's depth. However, potential weaknesses exist. These include the need for better control over female crab origin to minimize host condition variability, the qualitative nature of behavioral metrics that could be improved with quantitative tracking, and potentially low sample sizes in certain treatments, which might limit statistical power.

We agree that there are minor weaknesses in some elements of the methods. We have amended statements in the Methods, Results and Discussion to reflect this. However, core elements of the study stand as they present novel information and a foundation for future research to expand upon. For example, the low sample sizes are a feature of the hyposaline stress experiment (n=4-5 per treatment) and demonstrate a certain degree of resiliency by the worms depending on their exposure times. The sample sizes in the other experiments were fine (n=6 independent observations per treatment, with good survival) demonstrating the utility of the response metrics that we developed. We agree that quantitative tracking could potentially improve the behavioral metrics; however, it is not needed to derive the findings and conclusions from our work.

The hypotheses overlap significantly and could be reworded to eliminate redundancy. Both essentially propose that salinity is positively correlated with nemertean presence, with the first focusing on infestation probability and the second on presence and abundance.
To improve clarity, these hypotheses could be combined into a single statement:
"We hypothesize that salinity will be positively correlated with the presence, abundance, and probability of nemertean infestation, as these organisms are expected to thrive in euryhaline waters."

We have consolidated the hypothesis to reduce redundancy. However, presence, abundance and probability of infestation are all different metrics that may warrant separate hypotheses.

Host Collection: Please specify the original salinity/salinities.

This information has been added (line 115, line 181).

Lines 108, 170, and 295: When referring to "hyposaline stress," clarify which specific salinity levels were used.

This information has been added in two locations: the hyposaline recovery section of methods and results (lines 180 and 311 in revised document).

Line 117: Reword for formality: “containing approximately 3 mL.” same through the paper.

All “~” symbols have been replaced with “approximately”.

Line 120: Explain how the experimental water was prepared, including details on the device used to measure salinity.

A sentence detailing this has been added; see line 125-126 in revised document.

Line 139: Clarify the categorization of worm location within the petri dish to distinguish positional differences more explicitly. There is potential for subjectivity in this response.

A sentence clarifying different worm locations can be found at lines 145-146 (revised document). We agree with the reviewer that there is potential for subjectivity in this response. We have added worm body position and location to the sentence addressing subjectivity of qualitative metric to improve clarity and transparency. Other more objective metrics also showed consistent patterns.

Line 162: The phrase “when an individual exhibited one or more of these taxes” is unclear. consider rewording to “behavioral responses”. Repeated use of "healthy": Consider simplifying or defining the criteria concisely.

Line 160: We changed “healthy” to “good condition”. We also adopted the reviewer’s suggestion regarding behavioral responses.

Line 175: When mentioning crabs from the lower Chesapeake Bay, include the recorded salinity for the experiments.

This information has been added. (line 181, revised document)

Lines 183-184: Provide a reference to support the consideration of mucus production as a health indicator.

Line 190: Reference [1] (Humes, 1942) has been added to these statements. He made extensive comments regarding mucus production in his monograph.

Line 195: Specify the total number of ovigerous females examined.

This information has been added. (line 195, revised document)

Line 224: Include female as a random factor in the experimental design and modeling to account for individual variation.

We believe this comment is addressing the study’s model of nemertean presence and abundance. We are not including female as a random factor in the model because individual female (the presence and abundance of an individual) is the sampling unit. The variation of individual is our model output. We did consider including random effects, such as trawl tow, but due to some of the tow stations acquiring only 1 crab per tow, the model is unable to parse the variation due to small sample size.

Line 112: Indicate the number of female crabs used in the experiment.
Controlling Female Crab Origin: To ensure statistical and experimental consistency, the origin of female crabs should be explicitly stated in the experimental design. This would help control for potential variability in host condition and exposure history.

These comments are addressed collectively. (1) We have adjusted the wording in the salinity tolerance and hyposaline recovery methods sections to explicitly state that the worms were extracted from one female from each location, a total of two host females (one from Cobb Bay on the Eastern Shore, and one from the lower mainstem of the Bay).

(2) We began this experiment in late September, early October of 2022, near the very end of blue crab spawning season. Acquiring infected fecund females late in field season proved difficult, which contributed to our notably small sample of host females.

(3) We have added host origin as a factor that should be further explored to improve the statical significance of this study to a statement in the discussion (line 452, revised document). We recognize that worms from additional females would have improved the manuscript, and we had discussed this very aspect early in the concept for this work, but the late start affected our ability to obtain live female crabs for experimentation.

Editor’s comment: I would suggest to add more references and recents on the effect of salinity on the blue crab Callinectes sapidus to contextualised better your study.”

Although the ms is about the nemertean’s tolerance to different salinities, as per the Editor’s suggestion, we have added two additional references and a sentence in the Introduction regarding the salinity fluctuations experience by the blue crab host:

Marchessaux, G., Barré, N., Mauclert, V., Lombardini, K., Durieux, E.D., Veyssiere, D., Filippi, J.J., Bracconi, J., Aiello, A. and Garrido, M., 2024. Salinity tolerance of the invasive blue crab Callinectes sapidus: From global to local, a new tool for implementing management strategy. *Science of the Total Environment*, *954*, p.176291.

Jivoff, P.R., Smith, J.M., Sodi, V.L., Van Morter, S.M., Faugno, K.M., Werda, A.L., Shaw, J.J. 2017. Population Structure of Adult Blue Crabs, *Callinectes sapidus*, in Relation to Physical Characteristics in Barnegat Bay, New Jersey. *Estuaries and Coasts* **40**, 235–250 (2017). https://doi.org/10.1007/s12237-016-0127-8

**Journal Requirements:**

1. When submitting your revision, we need you to address these additional requirements.

Please ensure that your manuscript meets PLOS ONE's style requirements, including those for file naming. The PLOS ONE style templates can be found at

<https://journals.plos.org/plosone/s/file?id=wjVg/PLOSOne_formatting_sample_main_body.pdf> and

<https://journals.plos.org/plosone/s/file?id=ba62/PLOSOne_formatting_sample_title_authors_affiliations.pdf>

The manuscript has been formatted according to the above referenced pdf files.

2. Thank you for stating the following financial disclosure:

AKS, Willard A. Van Engel Fellowship at VIMS

AKS, Virginia Sea Grant #222030

AKP, REU Fellowship, NSF #1950242

Funding not specifically for this project, but for student support in educational training.

Please state what role the funders took in the study.  If the funders had no role, please state: "The funders had no role in study design, data collection and analysis, decision to publish, or preparation of the manuscript."

If this statement is not correct you must amend it as needed.

Please include this amended Role of Funder statement in your cover letter; we will change the online submission form on your behalf.

Noted. We have clarified this in the amended Cover Letter as well as here.

AKP received an NSF REU Fellowship (NSF #1950242 to R.D. Seitz) for summer internship support. This funding was not specifically for this project, but for student support in educational training. AKS received the Willard A. Van Engel Fellowship in support of her graduate training at VIMS, including her work on this project. AKS also received a Virginia Sea Grant Fellowship (#22203) in support of her graduate studies on the reproductive ecology of blue crabs, which included her work and supplies used for this project. The funders had no role in study design, data collection and analysis, decision to publish, or preparation of the manuscript.

3. Thank you for stating the following in the Acknowledgments Section of your manuscript:

We thank Professor Randy Chambers and Dr. Jenny Rahn for their guidance and support. The crew and captains of the VIMS Trawl Survey, particularly Wendy Lowery, helped by obtaining crabs. The VIMS trawl survey is funded by VIMS, The Virginia Marine Resources Commission, and U.S. Fish and Wildlife. Special thanks to Challen Hyman, Violet Johnston, Jainita Patel, Katie Knick, Mike Seebo, and the VIMS Community Ecology & Conservation Lab for their guidance and help with dissections. This work was supported in part by an NSF REU grant 1950242 to R.D. Seitz, Willard A. Van Engel Fellowship, and the Virginia Sea Grant Graduate Fellowship under Grant #V721500, Virginia Sea Grant College Program Project No. 222030, from the National Oceanic and Atmospheric Administration’s (NOAA) National Sea Grant College Program, U.S. Department of Commerce. The statements, findings, conclusions, and recommendations are those of the authors and do not necessarily reflect the views of Virginia Sea Grant, NOAA, or the U.S. Department of Commerce.

We note that you have provided funding information that is not currently declared in your Funding Statement. However, funding information should not appear in the Acknowledgments section or other areas of your manuscript. We will only publish funding information present in the Funding Statement section of the online submission form.

Please remove any funding-related text from the manuscript and let us know how you would like to update your Funding Statement. Currently, your Funding Statement reads as follows:

AKS, Willard A. Van Engel Fellowship at VIMS

AKS, Virginia Sea Grant #222030

AKP, REU Fellowship, NSF #1950242

Funding not specifically for this project, but for student support in educational training.

Please include your amended statements within your cover letter; we will change the online submission form on your behalf.

Duly noted. I believe I have addressed this fully in the previous section and in the amended cover letter. -jds

4. In the online submission form, you indicated that your data will be submitted to a repository upon acceptance.  We strongly recommend all authors deposit their data before acceptance, as the process can be lengthy and hold up publication timelines. Please note that, though access restrictions are acceptable now, your entire minimal dataset will need to be made freely accessible if your manuscript is accepted for publication. This policy applies to all data except where public deposition would breach compliance with the protocol approved by your research ethics board. If you are unable to adhere to our open data policy, please kindly revise your statement to explain your reasoning and we will seek the editor's input on an exemption.

We have uploaded our data to figshare at: [https://figshare.com/s/641d53ba42e7f369b6ae](https://nam11.safelinks.protection.outlook.com/?url=https%3A%2F%2Ffigshare.com%2Fs%2F641d53ba42e7f369b6ae&data=05%7C02%7Cjeff%40vims.edu%7Cfd4b37ff1f2040ca95ae08dd9e21716b%7C8cbcddd9588d4e3b9c1e2367dbdf1740%7C0%7C1%7C638840589166813067%7CUnknown%7CTWFpbGZsb3d8eyJFbXB0eU1hcGkiOnRydWUsIlYiOiIwLjAuMDAwMCIsIlAiOiJXaW4zMiIsIkFOIjoiTWFpbCIsIldUIjoyfQ%3D%3D%7C0%7C%7C%7C&sdata=VdhmXHydQuR2iLJ32oADoGiZB1AXvJKbLm5h2lyqllY%3D&reserved=0)

5. When completing the data availability statement of the submission form, you indicated that you will make your data available on acceptance. We strongly recommend all authors decide on a data sharing plan before acceptance, as the process can be lengthy and hold up publication timelines. Please note that, though access restrictions are acceptable now, your entire data will need to be made freely accessible if your manuscript is accepted for publication. This policy applies to all data except where public deposition would breach compliance with the protocol approved by your research ethics board. If you are unable to adhere to our open data policy, please kindly revise your statement to explain your reasoning and we will seek the editor's input on an exemption. Please be assured that, once you have provided your new statement, the assessment of your exemption will not hold up the peer review process.

As noted above, data files are at figshare: [https://figshare.com/s/641d53ba42e7f369b6ae](https://nam11.safelinks.protection.outlook.com/?url=https%3A%2F%2Ffigshare.com%2Fs%2F641d53ba42e7f369b6ae&data=05%7C02%7Cjeff%40vims.edu%7Cfd4b37ff1f2040ca95ae08dd9e21716b%7C8cbcddd9588d4e3b9c1e2367dbdf1740%7C0%7C1%7C638840589166813067%7CUnknown%7CTWFpbGZsb3d8eyJFbXB0eU1hcGkiOnRydWUsIlYiOiIwLjAuMDAwMCIsIlAiOiJXaW4zMiIsIkFOIjoiTWFpbCIsIldUIjoyfQ%3D%3D%7C0%7C%7C%7C&sdata=VdhmXHydQuR2iLJ32oADoGiZB1AXvJKbLm5h2lyqllY%3D&reserved=0)

6. Please include your full ethics statement in the ‘Methods’ section of your manuscript file. In your statement, please include the full name of the IRB or ethics committee who approved or waived your study, as well as whether or not you obtained informed written or verbal consent. If consent was waived for your study, please include this information in your statement as well.

Line 268 or thereabouts. **Ethics statement**: This research did not require review by institutional review boards.

7. We note that Figures 4 and 5 in your submission contain map images which may be copyrighted. All PLOS content is published under the Creative Commons Attribution License (CC BY 4.0), which means that the manuscript, images, and Supporting Information files will be freely available online, and any third party is permitted to access, download, copy, distribute, and use these materials in any way, even commercially, with proper attribution. For these reasons, we cannot publish previously copyrighted maps or satellite images created using proprietary data, such as Google software (Google Maps, Street View, and Earth). For more information, see our copyright guidelines: <http://journals.plos.org/plosone/s/licenses-and-copyright>.

We require you to either present written permission from the copyright holder to publish these figures specifically under the CC BY 4.0 license, or remove the figures from your submission:

**Figures 4 and 5 were generated as shapefiles in R and do not require copyright permissions.**
